# Imatinib Regulates *miR-483-3p* and Mitochondrial Respiratory Complexes in Gastrointestinal Stromal Tumors

**DOI:** 10.3390/ijms221910600

**Published:** 2021-09-30

**Authors:** Wen-Kuan Huang, Hao Shi, Pinar Akçakaya, Katarina Zeljic, Anastasia Gangaev, Stefano Caramuta, Chun-Nan Yeh, Robert Bränström, Catharina Larsson, Weng-Onn Lui

**Affiliations:** 1 Department of Oncology-Pathology, Karolinska Institutet, BioClinicum J6:20, Karolinska University Hospital, 171 64 Solna, Sweden; hao.shi@ki.se (H.S.); pinarakcakaya@gmail.com (P.A.); katarina.zeljic@bio.bg.ac.rs (K.Z.); a.gangaev@nki.nl (A.G.); stefano.caramuta@ucr.uu.se (S.C.); catharina.larsson@ki.se (C.L.); 2 Division of Hematology-Oncology, Department of Internal Medicine, Chang Gung Memorial Hospital at Linkou, Taoyuan 33305, Taiwan; 3 Department of Surgery, Chang Gung Memorial Hospital and GIST Team at Linkou, Chang Gung University College of Medicine, Taoyuan 33305, Taiwan; yehchunnan@gmail.com; 4 Department of Molecular Medicine and Surgery, Karolinska Institutet, 171 76 Stockholm, Sweden; robert.branstrom@ki.se

**Keywords:** gastrointestinal stromal tumor (GIST), imatinib, microRNA, oxidative phosphorylation, *miR-483*, succinate dehydrogenase B, Complex II

## Abstract

Metabolic adaptation to increased oxidative phosphorylation (OXPHOS) has been found in gastrointestinal stromal tumor (GIST) upon imatinib treatment. However, the underlying mechanism of imatinib-induced OXPHOS is unknown. Discovering molecules that mediate imatinib-induced OXPHOS may lead to the development of therapeutic strategies synergizing the efficacy of imatinib. In this study, we explored the role of microRNAs in regulating OXPHOS in GIST upon imatinib treatment. Using a microarray approach, we found that *miR-483-3p* was one of the most downregulated miRNAs in imatinib-treated tumors compared to untreated tumors. Using an extended series of GIST samples, we further validated the downregulation of *miR-483-3p* in imatinib-treated GIST samples by RT-qPCR. Using both gain- and loss-of-function experiments, we showed that *miR-483-3p* could regulate mitochondrial respiratory Complex II expression, suggesting its role in OXPHOS regulation. Functionally, *miR-483-3p* overexpression could rescue imatinib-induced cell death. These findings provide the molecular link for imatinib-induced OXPHOS expression and the biological role of *miR-483-3p* in regulating cell viability upon imatinib treatment.

## 1. Introduction

Gastrointestinal stromal tumor (GIST) is the most common type of gastrointestinal mesenchymal tumor, primarily harboring gain of function mutations in the *KIT* or platelet-derived growth factor receptor-alpha (*PDGFRA*) genes [1]. The tyrosine kinase inhibitor imatinib that blocks c-KIT and PDGFRA activation is the mainstay treatment for advanced GIST, which has led to improved survival with a median progression-free survival of 18–20 months and a median overall survival of 52–72 months [2,3,4,5]. However, most patients who initially respond to imatinib treatment develop acquired resistance and tumor progression after two years [6]. 

Deregulated cell metabolism has been recognized as a hallmark of cancer [7]. Although the Warburg effect is generally accepted as the synonym of metabolic reprogramming, recent emerging evidence disclosed that tumor cells could alternate their bioenergetics between glycolysis and oxidative phosphorylation (OXPHOS) in a context-dependent fashion [8]. Metabolic adaptation with shifting from glycolysis to OXPHOS has been reported to promote cancer cell survival under the stress of drug treatment [9,10]. As shown by us [11] and Vitiello et al. [12], GIST cells can upregulate OXPHOS levels of Complex II, III, and V as an adaptation to imatinib treatment. Targeting OXPHOS enhances the cytotoxic effect of imatinib in GIST [12]. 

The underlying mechanism of metabolic reprogramming upon imatinib treatment in GIST remains elusive. We previously demonstrated that imatinib also suppressed mitochondrial biogenesis [11], suggesting that imatinib does not induce OXPHOS through mitochondrial biogenesis. In the present study, we speculate that imatinib might induce OXPHOS through microRNA regulation in GIST.

MicroRNAs (miRNAs) are small non-coding RNA molecules with a length of 18–25 nucleotides that regulate gene expression in a sequence-specific manner. Several miRNAs have been demonstrated to regulate OXPHOS by targeting the expression of mitochondrial electron transport chain complexes. For example, *miR-210* has been demonstrated to target multiple mitochondrial respiratory complexes, including Complex I (*NDUFA4*) [13,14], Complex II (*SDHD*) [14], and Complex IV (*COX10*) [15]. Both *miR-181c* and *miR-338* can target Complex IV (*COX1* [16] and *COX4* [17], respectively), while *miR-127-5p* and *miR-141* regulate Complex V (ATP synthases) [18,19].

Herein, we compared miRNA expression profiles between imatinib-treated and untreated GIST tumors and identified a subset of miRNAs that were differentially expressed between the two groups. Among them, *miR-483-3p* was further investigated for its association with imatinib treatment and its involvement in OXPHOS regulation. Our results suggest that *miR-483-3p* may contribute to imatinib-induced OXPHOS in GIST cells.

## 2. Results

### 2.1. Differentially Expressed miRNAs between Imatinib-Treated and Untreated GISTs 

Global miRNA expression levels of 34 GISTs, including 19 imatinib-treated and 15 untreated tumors, were characterized using a microarray-based platform. After normalization and filtering, 139 miRNAs were included for further analyses. Using SAM analysis, 12 up-regulated and 2 down-regulated miRNAs were identified with a false discovery rate (FDR, q-value) of <20% in the imatinib-treated compared to untreated GISTs (Table 1).

**Figure 1:**
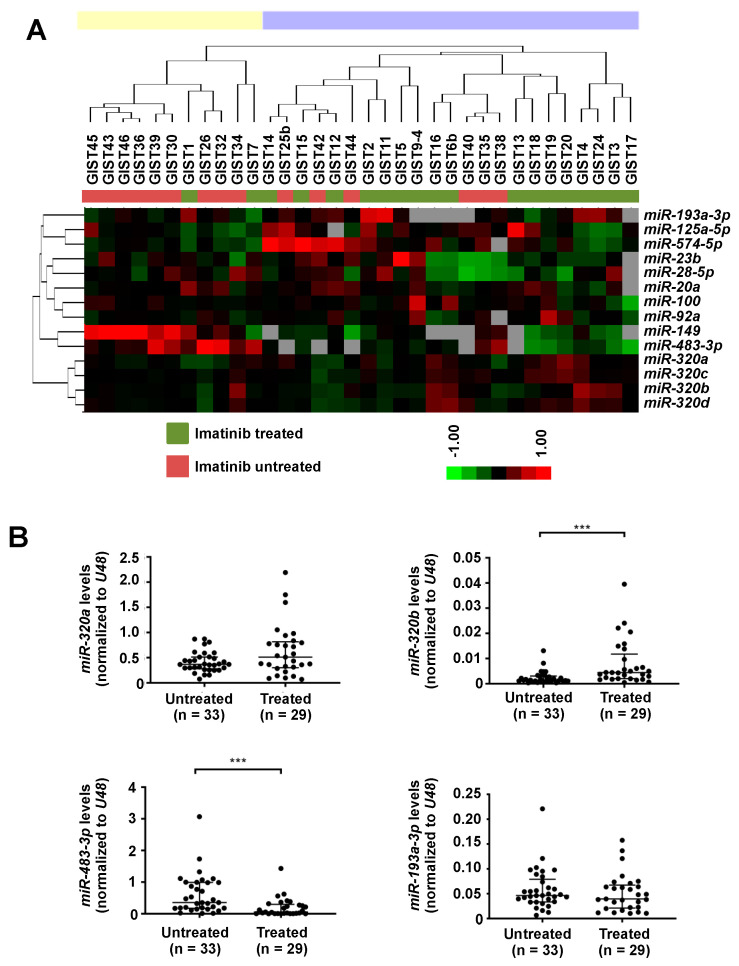
miRNA expressions in imatinib-treated and untreated gastrointestinal stromal tumor (GIST) samples. (**A**) Hierarchical clustering of the 14 differentially expressed miRNAs between imatinib-treated (*n* = 19) and untreated GISTs (*n* = 15) was performed based on the Spearman’s Rank Order Correlation and complete linkage. Red and green in the heatmap refer to higher and lower expressions, respectively. The two main clusters are indicated by yellow and blue bars above the dendrogram. (**B**) RT-qPCR analysis of four selected differentially expressed miRNAs in an extended cohort of 33 untreated and 29 treated GIST samples. *** *p* < 0.001 by Mann–Whitney U-test.

We then performed hierarchical clustering analysis of the 34 GIST samples using these 14 significant differentially expressed miRNAs. The analysis classified the samples into two main clusters: all imatinib untreated samples but two (GIST1 and GIST7) were grouped together, while the majority of the treated samples (17 out of 19) were clustered in a separate group (Figure 1A).

To validate the miRNA array data, we performed RT-qPCR analyses of four selected miRNAs in an extended cohort of 62 samples. We found significantly higher expression of *miR-320b* (*p* = 0.001, Mann–Whitney U-test) and lower expression of *miR-483-3p* (*p* < 0.001, Mann Whitney U-test) in imatinib-treated GISTs, as compared to untreated GISTs (Figure 1B). The expression of *miR-320a* and *miR-193a-3p* did not differ significantly between imatinib-treated and untreated tumors (Figure 1B).

### 2.2. miR-320b and miR-483-3p Expression in GIST Cells upon Imatinib Treatment

**Figure 2:**
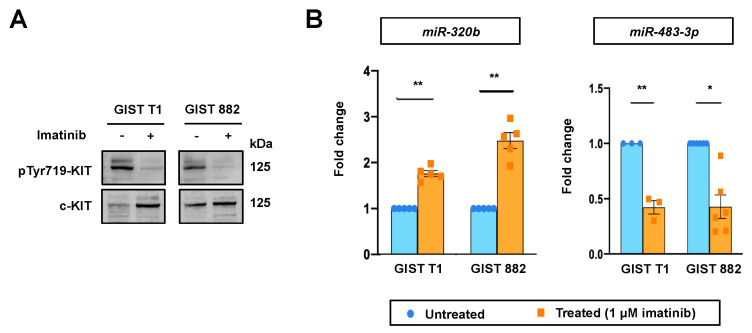
Effect of imatinib treatment on miRNA expressions in GIST cell lines. (**A**) Western blot showing the effectiveness of imatinib treatment in GIST cell lines, as demonstrated by inhibition of KIT phosphorylation. (**B**) RT-qPCR analyses of *miR-320b* and *miR-483-3p* were performed in GIST T1 and GIST 882 cell lines after imatinib treatment (1 μM, 24 h). miRNA expression was normalized to *U48*. Fold change refers to the normalized miRNA expression values relative to their respective untreated cells. Differences between groups were calculated using paired *t*-test. * *p* < 0.05, ** *p* < 0.01.

To determine whether *miR-320b* and *miR-483-3p* can be regulated by imatinib, we quantified their expressions in GIST cell lines after 24 h of imatinib treatment. Consistent with the observations in clinical samples, we found significant upregulation of *miR-320b* and downregulation of *miR-483-3p* in GIST 882 and GIST T1 upon treatment with 1 μM imatinib (Figure 2).

### 2.3. Effects of miR-483-3p on OXPHOS Protein Expressions

**Figure 3:**
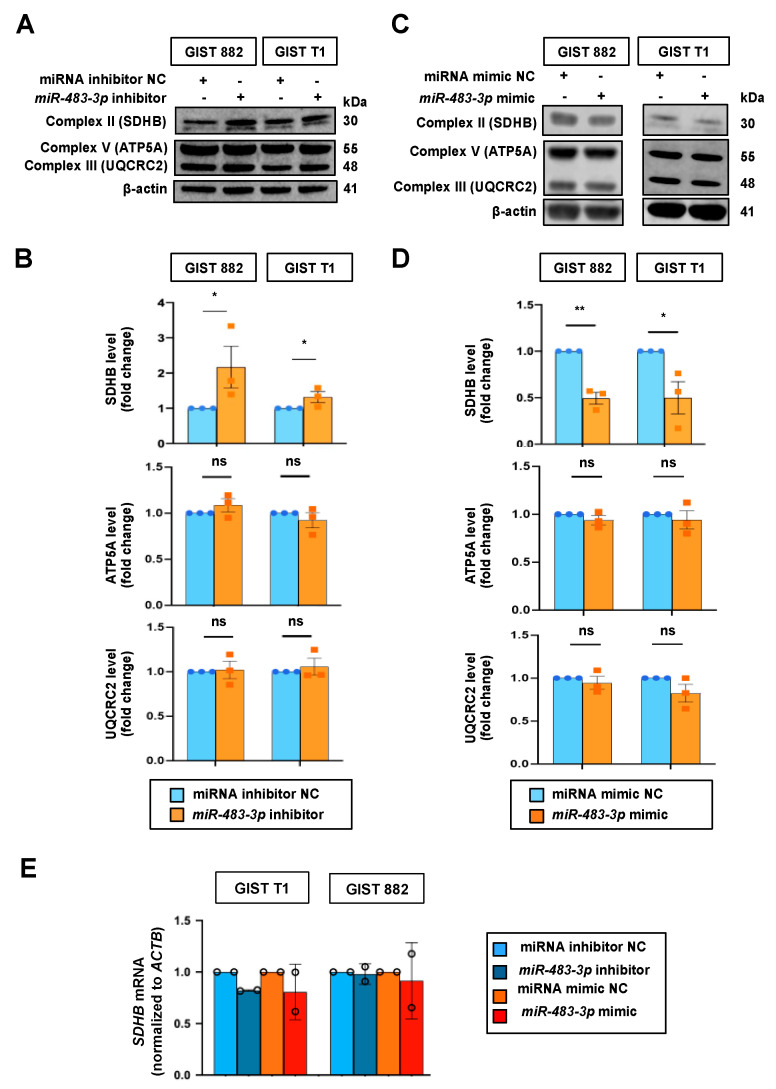
Effect of *miR-483-3p* overexpression and inhibition on oxidative phosphorylation (OXPHOS) protein expressions. (**A**,**C**) Representative Western blots showing OXPHOS proteins in GIST cell lines upon inhibition (**A**) or overexpression (**C**) of *miR-483-3p* and their respective non-targeting controls (NC) at 72 h post-transfection. Estimated protein sizes are given to the right of the blots in kiloDalton (kDa). (**B**,**D**) Bar plots showing quantification of OXPHOS proteins in (**A**,**C**). β-actin was used as a loading control. miRNA inhibitor and mimic non-targeting controls were set to 1.0. (**E**) RT-qPCR analysis of *SDHB* mRNA in GIST cells with overexpression or inhibition of *miR-483-3p*. Error bars refer to SD of two or three independent experiments. Differences between groups were calculated using paired *t*-test. * *p* < 0.05, ** *p* < 0.01, ns = not significant.

Vitiello et al. and our previous study have shown upregulated OXPHOS proteins upon imatinib treatment [11,12]. Given that *miR-483-3p* is involved in the insulin pathway and in glucose metabolism [20], we further investigated whether *miR-483-3p* plays a role in the regulation of OXPHOS proteins (Figure 3). In both GIST cell lines, we observed that inhibition of *miR-483-3p*, using miRNA inhibitor, increased protein expression of Complex II (SDHB), but not Complexes III (UQCRC2) or V (ATP5A) (Figure 3A,B). Conversely, overexpression of *miR-483-3p*, using miRNA mimic, reduced Complex II (SDHB) protein levels in both cell lines, while no significant changes were observed for Complex III (UQCRC2) or V (ATP5A) (Figure 3C,D). Notably, *miR-483-3p* overexpression or inhibition did not yield significant changes in *SDHB* mRNA levels (Figure 3E). 

### 2.4. Relationship between miR-483-3p and SDHB Expression upon Imatinib Treatment

**Figure 4:**
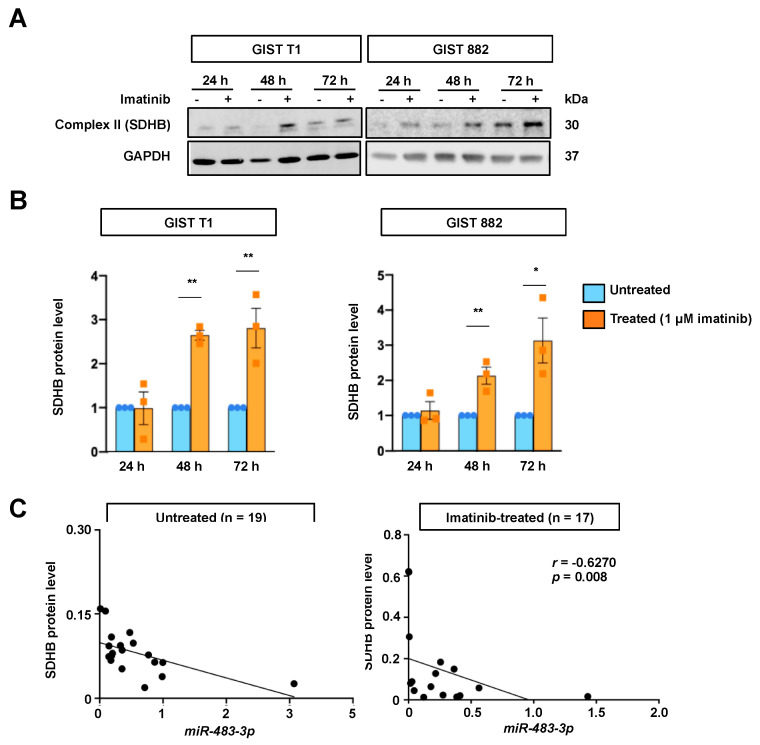
Relationship between succinate dehydrogenase B (SDHB) protein expression and imatinib treatment or *miR-483-3p*. (**A**) Representative Western blots showing SDHB protein expression in GIST cell lines with and without imatinib treatment (1 μM). GAPDH was used as a loading control. (**B**) Quantification of SDHB expression upon imatinib treatment of GIST cell lines at different time points. Differences between groups were calculated using paired *t*-test. * *p* < 0.05, ** *p* < 0.01 (*n* = 3). (**C**) Inverse correlation between SDHB protein expression and *miR-483-3p* in untreated and imatinib-treated GIST tumor samples.

Given that imatinib decreased *miR-483-3p* expression, we evaluated the expression of the Complex II component SDHB in GIST cell lines with and without imatinib treatment. As shown in Figure 4A,B, we observed increased SDHB protein levels in both cell lines after imatinib treatment for 48 h and 96 h. To further establish the relationship between *miR-483-3p* and SDHB expression, we compared *miR-483-3p* and SDHB protein expression in GIST tumor samples. The results showed inverse correlations between *miR-483-3p* and SDHB expression for both untreated (r = −0.5801, *p* = 0.009) and imatinib-treated (r = −0.6270, *p* = 0.008) GISTs (Figure 4C).

### 2.5. Functional Role of miR-483-3p in Regulation of Cell Viability upon Imatinib Treatment

**Figure 5:**
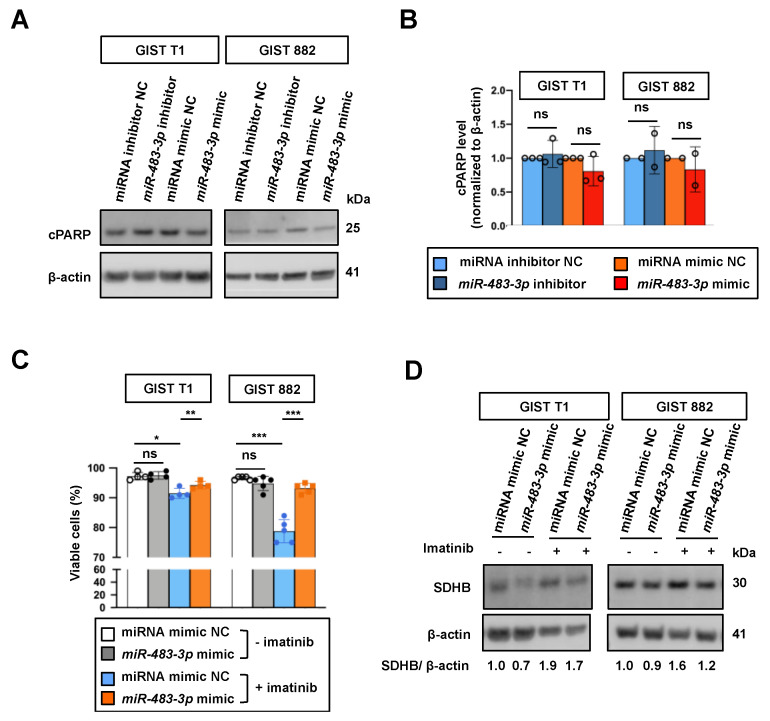
Effect of *miR-483-3p* regulation on cell viability with and without imatinib treatment. (**A**) Western blots showing the effect of *miR-483-3p* inhibition or overexpression on cleaved PARP (cPARP) expression in GIST cell lines. β-actin was used as a loading control. (**B**) Quantification of cPARP protein level in (**A**). (**C**) GIST cells were transfected with *miR-483-3p* mimic or NC for 24 h, followed by imatinib treatment for another 24 h. Cell viability was assessed by trypan blue staining. (**D**) Western blots showing the effect of *miR-483-3p* overexpression on SDHB protein level after 48 h of imatinib treatment. * *p* < 0.05, ** *p* < 0.01, *** *p* < 0.001, ns = not significant by paired *t*-test. Error bars represent SD. Each replicate is depicted by a dot.

To address the functional role of *miR-483-3p* in GIST cells, we assessed the effect of *miR-483-3p* inhibition or overexpression on the expression of cleaved PARP (cPARP), which is a marker for apoptosis. No significant changes were found on cPARP level upon inhibition or overexpression of *miR-483-3p* (Figure 5A,B), as well as cell viability in *miR-483-3p* overexpressing cells (Figure 5C). However, overexpression of *miR-483-3p* rescued cell death in imatinib treated cells, as assessed by trypan blue staining (Figure 5C). Notably, overexpression of *miR-483-3p* also reversed imatinib-induced SDHB expression (Figure 5D). Together, these results support the involvement of *miR-483-3p* in imatinib-induced SDHB expression and the important role of this regulation in protecting cell death induced by imatinib treatment.

## 3. Discussion

Cancer cells can exhibit metabolic adaptation in response to targeted therapy [21]. Several pieces of evidence have revealed that mitochondrial OXPHOS drives resistance to chemotherapy or targeted drugs in leukemia and solid tumors [22]. Similarly, GIST cells upregulate OXPHOS upon imatinib treatment [11,12]. However, the mechanism underlying such metabolic reprogramming is not fully understood. Here we propose the involvement of *miR-483-3p* in OXPHOS regulation in response to imatinib.

*miR-483* is located in intron 7 of the insulin-like growth factor 2 gene (*IGF2*) in chromosomal region 11p15. Two mature miRNAs, *miR-483-3p* and *miR-483-5p*, are processed from the stem-loop structure of the precursor and both are known to play important roles in cancer development [23]. *miR-483-3p* is often aberrantly overexpressed in cancers such as adrenocortical carcinoma [24], pancreatic cancer [25], Wilms’ tumor [26], hepatocellular carcinoma [27], colon cancer [26], and prostate cancer [28]. In contrast, downregulation of *miR-483-3p* has been found in some other cancer types, including nasopharyngeal cancer [29], breast [30], and gastric [31] cancers. These findings suggest that *miR-483-3p* may function as an oncogene or tumor suppressor, depending on the cellular context. In GIST, overexpression of *miR-483-3p* was found in CD117 positive/*KIT*-mutated tumors as compared to CD117 negative/*KIT*-unmutated tumors [32]. Wild-type GIST is a distinct subgroup of GIST that lacks *KIT* and *PDGFRA* mutations, which can be further classified as SDH-deficient or SDH-competent. SDH-deficient GISTs commonly harbor inactivating mutations in SDH subunit genes (*SDHA*, *SDHB*, *SDHC* or *SDHD*) or promoter hypermethylation of *SDHC* [33,34]. Increased expression of the insulin-like growth factor 1 receptor (IGF1R) is commonly found in SDH-deficient GISTs but rarely in SDH-competent GISTs [35]. Of note, IGF2 is a ligand exerting its effects by binding to the IGF1R. Future studies are warranted to investigate the relationship between *miR-483-3p*, IGF2, and SDH.

Here we observed reduced expression of *miR-483-3p* upon imatinib treatment. Although it is unclear how imatinib regulates *miR-483-3p* expression, *miR-483* transcription can be suppressed by glucose starvation and treatment with the glycolysis inhibitor 2-deoxy-d-glucose [36]. Given that imatinib can inhibit aerobic glycolysis and decrease glucose uptake [37], it is tempting to speculate that the reduction of *miR-483-3p* is caused by imatinib-mediated inhibition of glycolytic activity. The mechanism has yet to be elucidated.

Importantly, we demonstrate that *miR-483-3p* regulates Complex II but not Complexes III and V, suggesting the specificity of *miR-483-3p* in the regulation of Complex II. However, it is still unclear whether the regulation is direct or indirect. Complex II or SDH is a hetero-tetrameric protein complex consisting of SDHA, SDHB, SDHC and SDHD. Loss of any component of the SDH complex can lead to destabilization of the SDH complex and rapid degradation of the SDHB protein. Thus, loss of SDHB expression is a reliable marker for SDH deficiency [38,39], which leads to the question of which SDH subunit is a target of *miR-483-3p*. The interaction between *miR-483-3p* and SDH subunits is unknown. Although *SDHB* is not predicted as a target of *miR-483-3p*, *SDHC* is predicted as a putative target of *miR-483-3p* by RNAhybrid v2.2. Further investigation is needed to determine which SDH subunit is the potential direct target of *miR-483-3p*. To date, two miRNAs, *miR-147b* [40] and *miR-210* [13] have been reported to regulate SDHD. In our study, we did not find *miR-210* to be differentially expressed between imatinib-treated and untreated GIST samples, however, *miR-147b* was not detected.

Our previous work has shown that imatinib could increase the expression of several OXPHOS proteins, including Complexes II, III and V [11]. Given that *miR-483-3p* only regulates Complex II, it is possible that other miRNAs or factors could regulate OXPHOS proteins. It is worth mentioning that *miR-483-5p* was also downregulated in imatinib-treated as compared to untreated GIST tumors in our microarray data, however the FDR was 28.8%, which is higher than the arbitrary cut-off (20%) that we set in this study. This miRNA is predicted to target multiple genes involved in OXPHOS, including Complex I (*NDUFC1*, *NDUFA9*, *NDUFV3*, *NDUFA11*, and *NDUFA5*), Complex III (*UQCR11*), Complex IV (*COX19*, *COX6B1*) and Complex V (*ATP5S*, and *ATP5G1*) by TargetScanHuman release 7.2 [41]. The other downregulated miRNA, *miR-149*, has been demonstrated to target poly(ADP-ribose) polymerase-2 that activates the sirtuin-1/peroxisome proliferator–activated receptor γ coactivator-1α (PGC1α) pathway, leading to increased mitochondrial biogenesis [42]. The involvement of *miR-149* in mitochondrial biogenesis may provide the explanation for the suppression of PGC1α-dependent mitochondrial biogenesis by imatinib treatment observed in our previous study [11].

## 4. Conclusions

In conclusion, our results reveal that specific miRNAs are responsive to imatinib treatment. We also show that *miR-483-3p* can regulate mitochondrial respiratory Complex II, suggesting that suppression of this miRNA could partly contribute to increased OXPHOS upon imatinib treatment.

## 5. Materials and Methods 

### 5.1. Clinical Samples 

A total of 64 snap-frozen tumors from 56 GIST patients were included in this study. Of these, 30 tumors were from 24 patients who had received neoadjuvant imatinib treatment, and 34 tumors were from 32 patients who had not been treated with imatinib prior to surgery (referred to as untreated tumors). For 36 of the samples (19 untreated and 17 imatinib-treated), SDHB protein expression levels were previously determined by Western blot analysis as published in our previous study [11].

### 5.2. GIST Cell Lines and Imatinib Treatment 

Two imatinib-sensitive GIST cell lines were used, i.e., GIST 882 and GIST T1. GIST 882 was kindly provided by Dr. Jonathan Fletcher (Brigham and Women’s Hospital, Boston, MA, USA). This cell line was cultured in RPMI 1640 medium containing 15% fetal bovine serum, and 2 mM L-glutamine at 37 °C with 5% CO_2_. GIST T1 was purchased from Cosmo Bio Co., Ltd. (Tokyo, Japan) and cultured in DMEM supplemented with 10% fetal bovine serum. The authenticity of these cell lines was previously confirmed by short tandem repeat (STR) profiling [11]. GIST cell lines were treated with 1 μM imatinib for the indicated time periods. This imatinib concentration was according to previous publication [11]. 

### 5.3. RNA Extraction and Quantification

Total RNA was extracted with mirVana miRNA Isolation Kit (Thermo Fisher Scientific, Waltham, MA, USA) and RNA concentrations were measured using a NanoDrop spectrophotometer (NanoDrop Technologies, Wilmington, DE, USA). 

### 5.4. miRNA Expression Profiling 

Genome-wide miRNA expression profiles were determined for 19 imatinib-treated and 15 untreated tumors. Results for 17 of the imatinib-treated tumors have been previously reported [43]. All samples were analyzed using the human miRNA microarray system (Agilent, Santa Clara, CA, USA), which contains 903 human miRNAs (miRbase release 14). Array hybridizations and data analysis were according to the previously described methodology [44]. The data were normalized and centered to median using the Cluster 3.0 software (http://bonsai.hgc.jp/~mdehoon/software/cluster/software.htm, accessed on 2 November 2014). Normalized miRNAs with less than 20% of missing values were further analyzed using Significance Analysis of Microarrays (SAM) to identify differentially expressed miRNAs. Java TreeView software (http://jtreeview.sourceforge.net, accessed on 2 November 2014) was used to visualize the data from hierarchical clustering.

### 5.5. Reverse Transcription Quantitative PCR (RT-qPCR) 

Four selected mature miRNAs, including *miR-320a* (ID 002277), *miR-320b* (ID 002844), *miR-193a-3p* (ID 002250) and *miR-483-3p* (ID 002339), were quantified using TaqMan-based RT-qPCR assays (Thermo Fisher Scientific, Waltham, MA, USA). To obtain cDNA, total RNA (20 ng) was reverse-transcribed using TaqMan MicroRNA Reverse Transcription Kit (Thermo Fisher Scientific, Waltham, MA, USA). RT-qPCR was performed using the TaqMan Universal PCR Master Mix (Thermo Fisher Scientific, Waltham, MA, USA) in the ABI Prism 7900HT Real-time PCR System (Thermo Fisher Scientific, Waltham, MA, USA). *RNU48* (ID 001006) was used as an endogenous control. For quantification of *SDHB* mRNA, cDNA was synthesized from 100 ng total RNA using High Capacity cDNA Reverse Transcription kit (Thermo Fisher Scientific) and RT-qPCR was performed using TaqMan pre-designed gene expression assays *SDHB* (Hs01042482_m1) and *ACTB* (Hs01060665_g1). All reactions were performed in triplicate, and relative expression levels were calculated with the delta C_T_ method (2^−ΔCT^). For imatinib treatment experiments, the 2^−ΔCT^ values of treated cells were normalized to the untreated control cells.

### 5.6. In Vitro Transfection of GIST Cells and Cell Viability Analysis

mirVana *miR-483-3p* inhibitors and mimics, and their respective negative controls (mirVana miRNA mimic and inhibitor negative control#1), were purchased from Thermo Fisher Scientific (Waltham, MA, USA). A total of 3 × 10^6^ GIST T1 and 882 cells were transfected with 50 pmol of miRNA mimics or inhibitors using Amaxa Nucleofector (Lonza, Basel, Switzerland), and program X01 and T20, respectively, according to the manufacturer’s instructions. For Western blotting, cells were harvested after 72 h of transfection.

For cell viability analysis, cells at 24 h post-transfection were treated with or without 1 μM imatinib for another 24 h, followed by staining with 0.4% trypan blue stain (Thermo Fisher Scientific, Waltham, MA, USA). Viable cells were counted using the TC10 automatic cell counter (Bio-Rad).

### 5.7. Western Blot Analysis 

GIST cells were homogenized in NP-40 Lysis buffer (Thermo Fisher Scientific, Waltham, MA, USA) with the addition of 1% protease inhibitor (Sigma-Aldrich, St. Louis, MO, USA), 1 mM phenylmethanesulfonyl fluoride (Sigma-Aldrich, St. Louis, MO, USA) and 1% phosphatase inhibitor cocktail (Sigma-Aldrich, St. Louis, MO, USA). Protein concentrations were measured using Pierce BCA Protein Assay Kit (Thermo Fisher Scientific, Waltham, MA, USA). Thirty to fifty micrograms of lysates were separated by 4–12% NuPAGE^®^ Bis-Tris gels (Thermo Fisher Scientific, Waltham, MA, USA) and transferred to nitrocellulose membranes (Thermo Fisher Scientific, Waltham, MA, USA) at 4 °C with constant 35 V for 2 h. After blocking with 5% bovine serum albumin diluted in TBS with 0.5% Tween-20, membranes were incubated with primary antibody anti-pTyr719-KIT (#3391, Cell Signalling Technology, Danvers, MA, USA) at 1:1000, anti-KIT (#3308, Cell Signalling Technology, Danvers, MA, USA) at 1:1000, anti-SDHB (10620-1-AP, Proteintech) at dilution 1:1000, anti-ATP5A (ab14748, abcam) at 1:1000 or anti-UQCRC2 (14742-1-AP, Proteintech) at 1:1000. For normalization purpose, the membranes were incubated with anti-β-actin (A2228, Sigma-Aldrich, St. Louis, MO, USA) at 1:2000 or anti-GAPDH (#2118, Cell Signalling Technology, Danvers, MA, USA) at 1:2000. The secondary antibodies included anti-rabbit IRDye 680 (#926-68071, LI-COR, Lincoln, NE, USA) at dilution 1:10,000 or anti-mouse IRDye 680 (#926-32210, LI-COR, Lincoln, NE, USA) at 1:10000. Precision Plus Protein All Blue Standards (#161-0373; Bio-Rad Laboratories, Hercules, CA, USA) was used as an indicator of molecular weights. Detection was carried out with the Odyssey CLx scanner (LI-COR Biotechnology, Lincoln, NE, USA). Protein expression levels were quantified using the Odyssey ImageStudio Lite software (LI-COR Biotechnology, Lincoln, NE, USA). For analyses of expression levels in imatinib-treated cells, protein expression levels in treated cells were normalized to the levels in untreated control cells. 

### 5.8. Statistical Analysis 

All statistical analyses were performed using Graphpad Prism 8.0 (Graphpad software, La Jolla, CA, USA). Independent Student’s *t*-test or Mann-Whitney *U*-test was performed to compare the expression of miRNA and protein in different patient groups and cell lines. Paired *t*-test was used to compare two different conditions between the experimental groups. Correlation between *miR-483-3p* and SDHB protein levels was assessed by Spearman’s Rank Order Correlation. All *p*-values were based on two-tailed tests, and a *p*-value < 0.05 was considered as statistically significant. 

## Figures and Tables

**Table 1:** Microarray-based SAM results for top miRNAs in imatinib-treated (*n* = 19) vs. untreated (*n* = 15) tumors.

miRNA	Score (d)	FDR q-Value (%)
***Over-expressed in imatinib-treated GISTs***		
*hsa-miR-320a*	2.02	0.00
*hsa-miR-193a-3p*	1.87	0.00
*hsa-miR-320d*	1.80	0.00
*hsa-miR-320c*	1.76	0.00
*hsa-miR-320b*	1.44	12.33
*hsa-miR-574-5p*	1.42	12.33
*hsa-miR-23b*	1.38	12.33
*hsa-miR-125a-3p*	1.37	12.33
*hsa-miR-100*	1.34	12.33
*hsa-miR-20a*	1.34	12.33
*hsa-miR-28-5p*	1.30	12.33
*hsa-miR-92a*	1.30	12.33
***Under-expressed in imatinib-treated GISTs***		
*hsa-miR-149*	−2.69	0.00
*hsa-miR-483-3p*	−2.63	0.00

FDR = False discovery rate; only miRNAs with a q-value < 20% are listed.

## Data Availability

The microarray data are publicly available at the NCBI Gene Expression Omnibus (GSE63159).

## References

[1] Von Mehren M., Joensuu H. (2018). Gastrointestinal Stromal Tumors. J. Clin. Oncol..

[2] Blanke C.D., Demetri G.D., von Mehren M., Heinrich M.C., Eisenberg B., Fletcher J.A., Corless C.L., Fletcher C.D., Roberts P.J., Heinz D. (2008). Long-term results from a randomized phase II trial of standard- versus higher-dose imatinib mesylate for patients with unresectable or metastatic gastrointestinal stromal tumors expressing KIT. J. Clin. Oncol..

[3] Patrikidou A., Chabaud S., Ray-Coquard I., Bui B.N., Adenis A., Rios M., Bertucci F., Duffaud F., Chevreau C., Cupissol D. (2013). Influence of imatinib interruption and rechallenge on the residual disease in patients with advanced GIST: Results of the BFR14 prospective French Sarcoma Group randomised, phase III trial. Ann. Oncol..

[4] Gold J.S., van der Zwan S.M., Gonen M., Maki R.G., Singer S., Brennan M.F., Antonescu C.R., De Matteo R.P. (2007). Outcome of metastatic GIST in the era before tyrosine kinase inhibitors. Ann. Surg. Oncol..

[5] DeMatteo R.P., Lewis J.J., Leung D., Mudan S.S., Woodruff J.M., Brennan M.F. (2000). Two hundred gastrointestinal stromal tumors: Recurrence patterns and prognostic factors for survival. Ann. Surg..

[6] Chen P., Zong L., Zhao W., Shi L. (2010). Efficacy evaluation of imatinib treatment in patients with gastrointestinal stromal tumors: A meta-analysis. World J. Gastroenterol..

[7] Hanahan D., Weinberg R.A. (2011). Hallmarks of cancer: The next generation. Cell.

[8] Faubert B., Solmonson A., DeBerardinis R.J. (2020). Metabolic reprogramming and cancer progression. Science.

[9] Farge T., Saland E., de Toni F., Aroua N., Hosseini M., Perry R., Bosc C., Sugita M., Stuani L., Fraisse M. (2017). Chemotherapy-Resistant Human Acute Myeloid Leukemia Cells Are Not Enriched for Leukemic Stem Cells but Require Oxidative Metabolism. Cancer Discov..

[10] Hirpara J., Eu J.Q., Tan J.K.M., Wong A.L., Clement M.V., Kong L.R., Ohi N., Tsunoda T., Qu J., Goh B.C. (2019). Metabolic reprogramming of oncogene-addicted cancer cells to OXPHOS as a mechanism of drug resistance. Redox Biol..

[11] Huang W.K., Gao J., Chen Z., Shi H., Yuan J., Cui H.L., Yeh C.N., Branstrom R., Larsson C., Li S. (2020). Heterogeneity of Metabolic Vulnerability in Imatinib -Resistant Gastrointestinal Stromal Tumor. Cells.

[12] Vitiello G.A., Medina B.D., Zeng S., Bowler T.G., Zhang J.Q., Loo J.K., Param N.J., Liu M., Moral A.J., Zhao J.N. (2018). Mitochondrial Inhibition Augments the Efficacy of Imatinib by Resetting the Metabolic Phenotype of Gastrointestinal Stromal Tumor. Clin. Cancer Res..

[13] Puissegur M.P., Mazure N.M., Bertero T., Pradelli L., Grosso S., Robbe-Sermesant K., Maurin T., Lebrigand K., Cardinaud B., Hofman V. (2011). miR-210 is overexpressed in late stages of lung cancer and mediates mitochondrial alterations associated with modulation of HIF-1 activity. Cell Death Differ..

[14] Nakada C., Hijiya N., Tsukamoto Y., Yano S., Kai T., Uchida T., Kimoto M., Takahashi M., Daa T., Matsuura K. (2020). A transgenic mouse expressing miR-210 in proximal tubule cells shows mitochondrial alteration: Possible association of miR-210 with a shift in energy metabolism. J. Pathol..

[15] Chen Z., Li Y., Zhang H., Huang P., Luthra R. (2010). Hypoxia-regulated microRNA-210 modulates mitochondrial function and decreases ISCU and COX10 expression. Oncogene.

[16] Das S., Ferlito M., Kent O.A., Fox-Talbot K., Wang R., Liu D., Raghavachari N., Yang Y., Wheelan S.J., Murphy E. (2012). Nuclear miRNA regulates the mitochondrial genome in the heart. Circ. Res..

[17] Aschrafi A., Schwechter A.D., Mameza M.G., Natera-Naranjo O., Gioio A.E., Kaplan B.B. (2008). MicroRNA-338 regulates local cytochrome c oxidase IV mRNA levels and oxidative phosphorylation in the axons of sympathetic neurons. J. Neurosci..

[18] Willers I.M., Martinez-Reyes I., Martinez-Diez M., Cuezva J.M. (2012). miR-127-5p targets the 3’UTR of human beta-F1-ATPase mRNA and inhibits its translation. Biochim. Biophys. Acta.

[19] Baseler W.A., Thapa D., Jagannathan R., Dabkowski E.R., Croston T.L., Hollander J.M. (2012). miR-141 as a regulator of the mitochondrial phosphate carrier (Slc25a3) in the type 1 diabetic heart. Am. J. Physiol. Cell Physiol..

[20] Pepe F., Visone R., Veronese A. (2018). The Glucose-Regulated MiR-483-3p Influences Key Signaling Pathways in Cancer. Cancers.

[21] Sarmento-Ribeiro A.B., Scorilas A., Goncalves A.C., Efferth T., Trougakos I.P. (2019). The emergence of drug resistance to targeted cancer therapies: Clinical evidence. Drug Resist. Updates.

[22] Valcarcel-Jimenez L., Gaude E., Torrano V., Frezza C., Carracedo A. (2017). Mitochondrial Metabolism: Yin and Yang for Tumor Progression. Trends Endocrinol. Metab..

[23] Zhou W., Yang W., Ma J., Zhang H., Li Z., Zhang L., Liu J., Han Z., Wang H., Hong L. (2018). Role of miR-483 in digestive tract cancers: From basic research to clinical value. J. Cancer.

[24] Ozata D.M., Caramuta S., Velazquez-Fernandez D., Akcakaya P., Xie H., Hoog A., Zedenius J., Backdahl M., Larsson C., Lui W.O. (2011). The role of microRNA deregulation in the pathogenesis of adrenocortical carcinoma. Endocr. Relat. Cancer.

[25] Hao J., Zhang S., Zhou Y., Hu X., Shao C. (2011). MicroRNA 483-3p suppresses the expression of DPC4/Smad4 in pancreatic cancer. FEBS Lett..

[26] Veronese A., Lupini L., Consiglio J., Visone R., Ferracin M., Fornari F., Zanesi N., Alder H., D’Elia G., Gramantieri L. (2010). Oncogenic role of miR-483-3p at the IGF2/483 locus. Cancer Res..

[27] Veronese A., Visone R., Consiglio J., Acunzo M., Lupini L., Kim T., Ferracin M., Lovat F., Miotto E., Balatti V. (2011). Mutated beta-catenin evades a microRNA-dependent regulatory loop. Proc. Natl. Acad. Sci. USA.

[28] Korzeniewski N., Tosev G., Pahernik S., Hadaschik B., Hohenfellner M., Duensing S. (2015). Identification of cell-free microRNAs in the urine of patients with prostate cancer. Urol. Oncol..

[29] Yi C., Wang Q., Wang L., Huang Y., Li L., Liu L., Zhou X., Xie G., Kang T., Wang H. (2012). MiR-663, a microRNA targeting p21(WAF1/CIP1), promotes the proliferation and tumorigenesis of nasopharyngeal carcinoma. Oncogene.

[30] Menbari M.N., Rahimi K., Ahmadi A., Mohammadi-Yeganeh S., Elyasi A., Darvishi N., Hosseini V., Abdi M. (2020). miR-483-3p suppresses the proliferation and progression of human triple negative breast cancer cells by targeting the HDAC8>oncogene. J. Cell Physiol..

[31] Yu F.Y., Zhou C.Y., Liu Y.B., Wang B., Mao L., Li Y. (2018). miR-483 is down-regulated in gastric cancer and suppresses cell proliferation, invasion and protein O-GlcNAcylation by targeting OGT. Neoplasma.

[32] Wang Y., Li J., Kuang D., Wang X., Zhu Y., Xu S., Chen Y., Cheng H., Zhao Q., Duan Y. (2018). miR-148b-3p functions as a tumor suppressor in GISTs by directly targeting KIT. Cell Commun. Signal..

[33] Killian J.K., Miettinen M., Walker R.L., Wang Y., Zhu Y.J., Waterfall J.J., Noyes N., Retnakumar P., Yang Z., Smith W.I. (2014). Recurrent epimutation of SDHC in gastrointestinal stromal tumors. Sci. Transl. Med..

[34] Miettinen M., Lasota J. (2014). Succinate dehydrogenase deficient gastrointestinal stromal tumors (GISTs)-a review. Int. J. Biochem. Cell Biol..

[35] Lasota J., Wang Z., Kim S.Y., Helman L., Miettinen M. (2013). Expression of the receptor for type i insulin-like growth factor (IGF1R) in gastrointestinal stromal tumors: An immunohistochemical study of 1078 cases with diagnostic and therapeutic implications. Am. J. Surg. Pathol..

[36] Pepe F., Pagotto S., Soliman S., Rossi C., Lanuti P., Braconi C., Mariani-Costantini R., Visone R., Veronese A. (2017). Regulation of miR-483-3p by the O-linked N-acetylglucosamine transferase links chemosensitivity to glucose metabolism in liver cancer cells. Oncogenesis.

[37] Gottschalk S., Anderson N., Hainz C., Eckhardt S.G., Serkova N.J. (2004). Imatinib (STI571)-mediated changes in glucose metabolism in human leukemia BCR-ABL-positive cells. Clin. Cancer Res..

[38] van Nederveen F.H., Gaal J., Favier J., Korpershoek E., Oldenburg R.A., de Bruyn E.M., Sleddens H.F., Derkx P., Riviere J., Dannenberg H. (2009). An immunohistochemical procedure to detect patients with paraganglioma and phaeochromocytoma with germline SDHB, SDHC, or SDHD gene mutations: A retrospective and prospective analysis. Lancet Oncol..

[39] Gill A.J. (2018). Succinate dehydrogenase (SDH)-deficient neoplasia. Histopathology.

[40] Zhang W.C., Wells J.M., Chow K.H., Huang H., Yuan M., Saxena T., Melnick M.A., Politi K., Asara J.M., Costa D.B. (2019). miR-147b-mediated TCA cycle dysfunction and pseudohypoxia initiate drug tolerance to EGFR inhibitors in lung adenocarcinoma. Nat. Metab..

[41] Agarwal V., Bell G.W., Nam J.W., Bartel D.P. (2015). Predicting effective microRNA target sites in mammalian mRNAs. Elife.

[42] Mohamed J.S., Hajira A., Pardo P.S., Boriek A.M. (2014). MicroRNA-149 inhibits PARP-2 and promotes mitochondrial biogenesis via SIRT-1/PGC-1alpha network in skeletal muscle. Diabetes.

[43] Akcakaya P., Caramuta S., Ahlen J., Ghaderi M., Berglund E., Ostman A., Branstrom R., Larsson C., Lui W.O. (2014). microRNA expression signatures of gastrointestinal stromal tumours: Associations with imatinib resistance and patient outcome. Br. J. Cancer.

[44] Caramuta S., Egyhazi S., Rodolfo M., Witten D., Hansson J., Larsson C., Lui W.O. (2010). MicroRNA expression profiles associated with mutational status and survival in malignant melanoma. J. Investig. Dermatol..

